# Human genetic admixture

**DOI:** 10.1371/journal.pgen.1009374

**Published:** 2021-03-11

**Authors:** Katharine L. Korunes, Amy Goldberg

**Affiliations:** Department of Evolutionary Anthropology, Duke University, Durham, North Carolina, United States of America; HudsonAlpha Institute for Biotechnology, UNITED STATES

## Abstract

Throughout human history, large-scale migrations have facilitated the formation of populations with ancestry from multiple previously separated populations. This process leads to subsequent shuffling of genetic ancestry through recombination, producing variation in ancestry between populations, among individuals in a population, and along the genome within an individual. Recent methodological and empirical developments have elucidated the genomic signatures of this admixture process, bringing previously understudied admixed populations to the forefront of population and medical genetics. Under this theme, we present a collection of recent *PLOS Genetics* publications that exemplify recent progress in human genetic admixture studies, and we discuss potential areas for future work.

## Introduction

**Fig 1:**
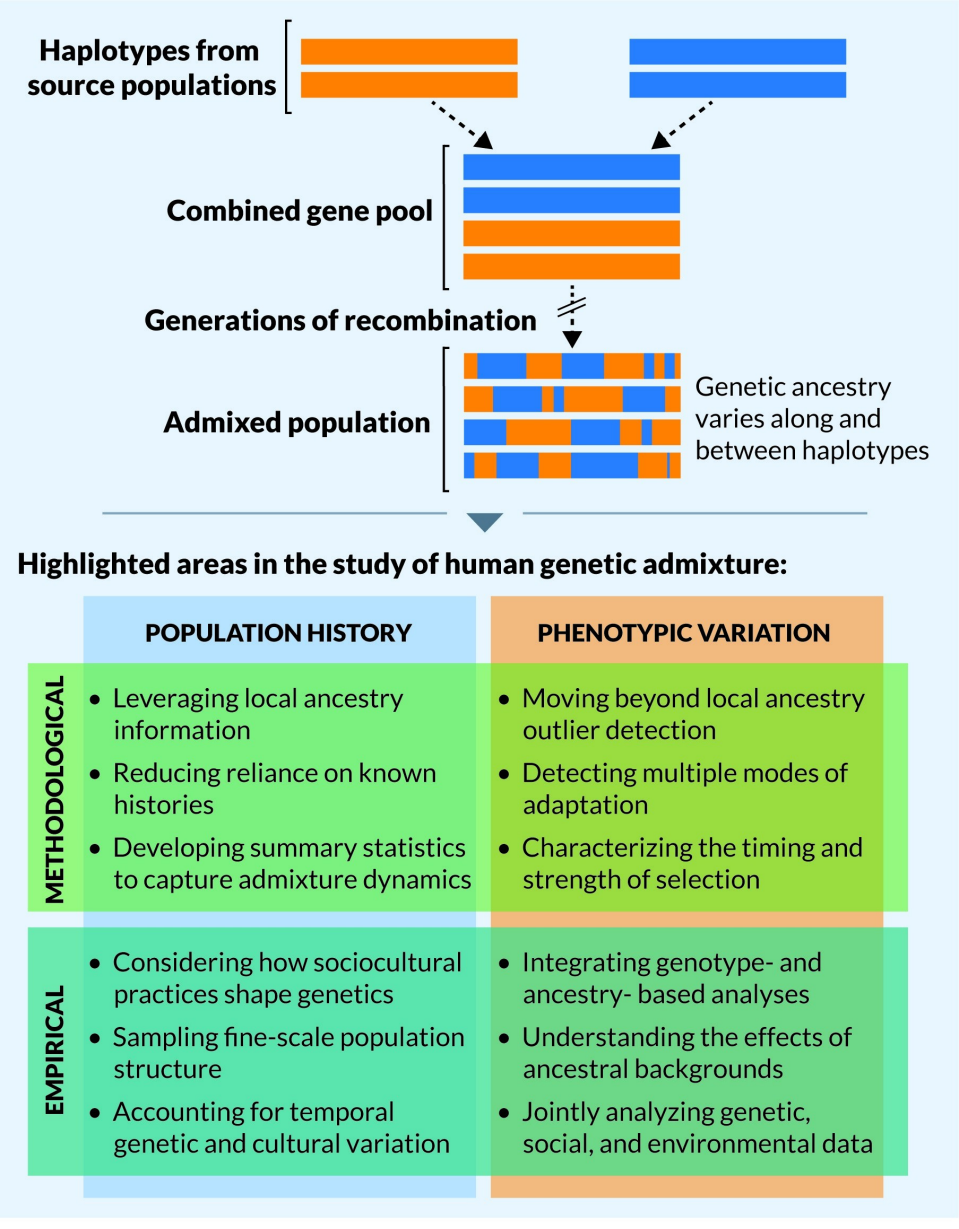
(Top) Large-scale movements of individuals allow haplotypes from previously isolated populations to come together in a combined gene pool. Generations of recombination between these haplotypes lead to an admixed population with genetic ancestry that varies between individuals and along haplotypes. The distribution of this variation is governed by the demographic and selection history of the admixed population and its sources. (Bottom) To leverage these patterns of admixed ancestry to better understand human history and phenotypic variation, we highlight key areas of recent progress and possible future directions in the study of human genetic admixture.

One of the major insights from the modern genomic era is the ubiquity of migration and admixture throughout human history [1–7]. Admixed populations are formed as moderate- to large-scale movements of individuals allow the exchange of genes from 2 or more previously isolated populations, creating populations with ancestors from multiple sources (Fig 1). These processes shape modern human genetic and phenotypic variation and may lead to differences in disease risk between populations [8–10]. Indeed, admixture is one of the fastest evolutionary processes to dramatically change the composition of a population. Despite their ubiquity and importance, admixed populations remain understudied in population and medical genetics [11–13], especially from a theoretical perspective. Recent empirical studies of admixed populations have emphasized inclusion of populations that have historically been excluded or underrepresented in genetic studies, producing important insights into human genetic and phenotypic variation.

Human population movement frequently lacks historical records. Many migration events have occurred through colonization or forced displacement, and ancient admixture often predates historical records. Therefore, genetic studies provide an opportunity to understand population history and the forces generating variation. Recent empirical work has shown that studying the genetics of a wider set of human populations can yield historical insights as well as medically relevant information about health and phenotypes. The mosaic ancestry patterns of admixed populations can also be used to elucidate the mechanisms and timescales of evolution in humans more generally. For example, ancestry patterns in admixed populations have been used to infer recombination rates [14–15] and to identify epistatically interacting [16–17] or adaptive [18–26] alleles. In this context, we use ancestry to describe “genetic ancestry”*—*the population origins of material within a genome. This is related to, but distinct, from genetic similarity and from genealogical ancestry, with the relationship further discussed by Mathieson and Scally [27].

Here, we highlight recent progress and discuss future directions for the study of admixed human populations (Fig 1). The term “admixture” encompasses multiple models of migration and population interactions; we focus on scenarios of human admixture with moderate to large contributions from at least 2 source populations, emphasizing the role that admixture has played throughout human evolution. Within this theme, we have curated a collection of *PLOS Genetics* publications. This compilation is a limited selection of work that exemplifies recent key advances and stimulates discussion about priorities for the future.

### Population history and demography

Broader genetic sampling of populations worldwide is increasingly being combined with advances in theory and computational methods to elucidate human history.

Genetic ancestry often varies along a chromosome within an individual and between individuals within an admixed population (Fig 1). Summaries of ancestry—such as the mean proportion of ancestry across individuals in a population, the proportion of ancestry at different genetic loci in a population, and the length of ancestry tracts within individuals—are shaped by the demographic history of a population. For example, drift may increase the variance in ancestry proportion within a population and across loci in small populations [28]. Similarly, recombination breaks up ancestry tracts over generations; therefore, more recent admixture events are expected to generally have longer tracts of a single ancestry within individuals.

Classic statistical methods in population genetics typically rely on allele frequencies, patterns of linkage disequilibrium (LD), and interpopulation sequence differences. However, the admixture process may distort patterns of LD, break up runs of homozygosity, and combine allele frequency distributions from distinct parental populations [28–29]. Yet, the patterns of genetic ancestry within an admixed population also provide another set of information about the history of a population. Theoretical work has sought to understand the relationship between demography and these summaries of genetic variation in admixed populations. Computational methods have built on this theory to infer population histories generating observed patterns of genetic ancestry [30–33]. Recent progress has focused on dynamic and complex population histories that may more accurately represent realistic population histories.

A number of major theoretical and methodological advances have recently been published in *PLOS Genetics* that improve our understanding of the demographic processes shaping genetic variation in admixed populations and human evolutionary history. Ragsdale and colleagues (2019) generalize 1- and 2-locus genetic summary statistics in a computationally efficient algorithm able to explore complex demographic models [34]. Other recent approaches focus on inferring population structure without known source populations or admixture history. The statistical method, SpaceMix, from Bradburd and colleagues (2016) can provide an intuitive visual summary of patterns of population structure and admixture by inferring a “geogenetic” map representing the geographic positions of the populations where distances describe rates of gene flow [35]. Methodological advances in applying LD and allele frequency summary statistics to admixed populations can clarify demographic histories even when reference genomes from contributing populations are unknown or poor quality. For example, the statistical method of Durvasula and Sankararaman (2019) combines several population genetic summary statistics to infer archaic ancestry along the genomes of modern individuals without relying on archaic reference genomes [36]. Identifying such segments of ancestry facilitates the use of methods that leverage local ancestry information. Advances in methods to study the demographic histories of admixed populations often leverage population- and genome-wide patterns of ancestry segments. For example, the IBD method of Browning and Browning (2015) was recently extended to capture ancestry-specific effective population size in admixed populations [37–38]. Browning and colleagues (2018) demonstrated a strategy for using identity-by-descent sharing in conjunction with local ancestry to detect fluctuations in historical population sizes [38]. Other methods leverage ancestral recombination graphs (ARGs), which can be used to trace coalescence and recombination events for each genomic position [39–41]. A recent application of ARGs by Hubisz and colleagues (2020) probabilistically samples ARGs to report probabilities of introgression along the genome [42].

Intricate sociocultural practices such as marriage customs, colonization events, and phenotypic preferences direct how parental populations interact to form admixed human populations. Whereas computational methods are increasingly considering these intricacies, empirical studies of admixed populations have already made substantial progress on fine-scale demography providing insight into how sociocultural practices shape the admixture process and within-region variation.

Using these new methods, empirical studies have brought to the forefront within-continent variation, particularly for historically excluded and under-sampled populations. Analyses of genomic ancestry within the context of geography have revealed between and within continent population structure [43]. For example, Moreno-Estrada and colleagues (2013) and Ruiz-Linares and colleagues (2014) show that the geographic distribution of admixture proportions in Latin America reflects complex demographic history and extensive admixture between Indigenous American, European, and African groups over Latin America’s history of peopling, colonization events, and forced displacement [44–45]. Recent studies of populations in the United States have also highlighted the complexities of admixture histories in the Americas [46–48]. Baharian and colleagues (2016) find that relatedness and ancestry patterns in African Americans show that the distribution of genetic diversity varies by region, reflecting differences in admixture history both before the Civil War and during the Great Migration in the 1900s [46]. Verdu and colleagues (2014) analyze genetic data from Indigenous communities in North America, finding different timing and sources of admixture compared to admixed populations of Central and South America, consistent with differences in historically documented migrations [47]. In Iran, Mehrjoo and colleagues (2019) found substantial population structure and variable levels of consanguinity across ethnic groups, highlighting the need to consider how population structure relates to cultural groups [49]. As cultural groups and customs within these groups shift over time, cultural transitions can further complicate the admixture process. For example, Martiniano and colleagues (2017) analyzed ancient genomes from Portugal to find changes in population structure, migration rates, and phenotypic predictions associated with Neolithic to Bronze Age cultural shifts [50].

Empirical studies such as these highlight the role social processes play in shaping genetic variation; for example, male and female demographic histories can differ. Considering population structure, sex-biased admixture, and effective population size changes, Font-Porterias and colleagues (2019) clarified the dynamic demographic history of European Roma groups, including showing that they share a common South Asian origin but have complex contributions from West Eurasian groups [51]. Another theme that surfaces in the populations highlighted above is the role of population structure and nonrandom mating in shaping the genetics of admixed populations. Empirical analyses of mating pairs have documented nonrandom pairings with respect to genetic ancestry, socioeconomic factors, and phenotypes, referred to as assortative mating [32,52–55]. Assortative mating has also been hypothesized to cause geographic structure in patterns of genetic and phenotypic variation in admixed populations [45]. Despite this complexity, methods often consider admixed populations as simple linear combinations of their parental populations. Moving forward, these empirical studies will inform the development of methods that account for social structure, as well as variation in the admixture history of a population over time and across geography. In turn, such models will improve inference of demographic history and neutral processes shaping diversity and act as better null models for selection scans and identification of phenotypically important loci.

### Phenotypically important loci and regions under selection

Selective pressures, such as those from environments and pathogens, play an important role in genetic variation and disease risk. Admixture both obscures genetic signatures of selection in source populations and provides new genetic material upon which selection can rapidly act.

Despite major progress on theory and methods to study demography in admixed human populations, methodological advances to study other processes such as adaptation and phenotypic variation remains an open area with substantial room for growth. Recent admixture may obscure genetic signatures of selection in the source populations by distorting linkage, rapidly changing allele frequencies, and breaking up homozygosity [28,56]. Therefore, current methods, which were built and tested on homogeneous populations, are underpowered and difficult to interpret. Although challenging, studying selection and identifying phenotypically important loci in admixed populations can provide a unique window into evolutionary processes. For example, admixed populations may provide pathways to studying genetic variation in populations that no longer exist in unadmixed form today, such as many Indigenous populations in the Americas. Similarly, admixed populations can be leveraged to increase sample sizes for understudied populations, for example, using African Americans to study West African evolutionary history. This is a critical area of current work, as much of our knowledge of the genetic basis of phenotypically diverse human traits is based on studies consisting overwhelmingly of individuals of European ancestry [11,57,58].

A common approach to identify candidate regions under selection post-admixture looks for outliers in local ancestry [18–21,59,60]. That is, regions of the genome with a higher frequency of ancestry from one source populations than genome-wide patterns are hypothesized to be enriched for genes under selection, as selection drives the haplotype of a single ancestry to higher frequency when the allele frequency of the locus differs substantially in the source populations. This approach has identified loci under strong recent selection. For example, the Duffy-null allele at the *DARC* locus is protective against malaria-causing *Plasmodium vivax* infection, which is estimated to be one of the strongest selective pressures in recent human history. The Duffy-null allele is nearly fixed in sub-Saharan African populations and mostly absent in non-African populations, producing a classic signal in which the *DARC* locus is an outlier in its proportion of local African ancestry in multiple admixed populations [21–25]. However, the strength of selection at the *DARC* locus is not typical of human adaptation. These local ancestry outlier approaches likely miss many loci under weaker or polygenic selection or those not highly differentiated in the sources. Additionally, outlier approaches generate false positives due to drift or long-range LD and discard other genetic information along the genome. For example, in Western African rainforest hunter–gatherer populations, Jarvis and colleagues (2012) found reduced levels of switching between ancestry types in a genomic region that may contribute to adaptive phenotypes such as short stature [26]. Despite the absence of outliers in local ancestry, multiple tests for selection corroborated evidence of local adaptation involving this region. New methods are needed to capture multiple modes of adaptation on the background of different demographic histories. Methods that leverage local genomic patterns and linkage may help to characterize the timing and strength of selection. In order to detect polygenic selection and understand quantitative trait variation, methods that leverage subtle shifts in ancestry across the genomes and GWAS in diverse populations may also be important.

Despite the challenges of identifying phenotypically important loci and regions under selection in admixed populations, recent empirical studies have highlighted the importance of using admixed populations to understand how selective pressures shape genetic variation and disease risk. Discovering phenotypically important loci in admixed populations requires careful consideration of the data, including strategies to integrate genotypes, local ancestry, and information from source populations. Once phenotypically important loci are identified, admixed populations can provide insight into how these loci interact with different ancestral backgrounds and how characteristics of the source populations shape post-admixture selection.

In addition to looking for correlations between genotype and phenotype, phenotypically important loci in admixed populations can be identified by looking for correlation between local ancestry and phenotype when the phenotype differs in the source populations [61]. A combination of these approaches can be used to understand quantitative trait variation in admixed populations. Beleza and colleagues (2013) provide one salient example of how genotype-based and ancestry-based association analyses can be integrated, in this case to understand the wide range of pigmentary phenotypic variation in skin and eyes in the admixed population of Cabo Verde [62]. This kind of integration of genotype- and ancestry- based approaches may help us understand how loci interact with different ancestral backgrounds. In admixed populations, local ancestry surrounding a known genetic risk factor can give clues about the other genetic variants that modulate that risk factor. For example, *ApoE* is the strongest known risk gene for late-onset Alzheimer disease; Rajabli and colleagues (2018) showed that variation in risk for Alzheimer disease across populations may be at least partially explained by the ancestral background interacting with the risk allele [63]. The relationship between phenotypes and genome-wide ancestry highlights the polygenic nature of many traits. Jeong and colleagues (2018) combined genomic and phenotypic data from Tibetan women from Nepal, adapted to high altitude, to find signatures of polygenic adaptation for traits involved in fertility and offspring survival [64]. In Latin American populations, Ruiz-Linares and colleagues (2014) jointly analyzed genetic variation, self-perceived ancestry, and a variety of physical traits, emphasizing the multifaceted relationship between genetic ancestry, social discussions of ancestry often forced into a classification system, and the genetic basis of traits. [45]. Going forward, it will be important to tease apart social and environmental factors that are often correlated with genetic ancestry and the genetic basis of complex traits.

Notably, these types of studies of quantitative variation and disease risk in admixed populations rely on careful consideration of the genotype and local ancestry calls, including reference-bias and characteristics of the source populations. Identifying phenotypically important loci in admixed populations is challenging in the absence of appropriate reference panels and knowledge of the source populations. The growing availability of genetic data from diverse groups shows that the unique LD structure of admixed groups and limited availability of suitable reference panels can particularly impair variant mapping and detection for novel or rare alleles. For example, Kowalski and colleagues (2019) recently analyzed a large cohort of phased genomes from African and Hispanic/Latino individuals (NHLBI TOPMed), revealing significant phenotypic associations for rare variants that were not detected with imputation using 1000 Genomes reference data [65].

Contributing source populations also have their own distinct selective and demographic histories. Although recent admixture obscures genetic signatures of selection in the source populations, consideration of past selective pressures can help identify important loci and clarify the genetic basis of disease risk. For example, Yao and colleagues (2018) found that in African ancestral backgrounds, past selection for the Duffy-null allele may also contribute to population differences in plasma levels of chemokines involved in a variety immune processes and diseases [66]. In Indigenous southern African populations, Chimusa and colleagues (2015) found signatures of selection both preceding and following inferred admixture events, including loci associated with selection imposed by diseases such as malaria, influenza, tuberculosis, and HIV/AIDS [67]. Considering the effective population sizes of source populations can also help explain how selection operates in an admixed population. Kim and colleagues (2018) used simulations of gene flow under various demographic scenarios and found that admixture can temporarily reduce genetic load in smaller populations, leading to an increase in the frequency of introgressed ancestry, particularly when existing and new deleterious mutations are recessive [68]. Relatedly, work from Juric and colleagues (2016) suggests that negative selection against Neanderthal sequences in the background of modern humans may be partially explained by Neanderthals having a much smaller effective population size compared to modern humans [69]. These examples demonstrate that understanding selection is closely related to demographic histories, including fluctuations in population size and migration. Future work inferring selection under complex demographies will be important, as well as further consideration of other modes of selection shaping variation in admixed populations, such as polygenic adaptation, background selection, and balancing selection.

## Conclusions

The set of papers highlighted here exemplifies recent advances and important areas for future work in the study of genetic admixture and its roles in human evolution. Recent theoretical and methodological advances have improved our understanding of the dynamic and complex demographic histories of modern human populations. New empirical insights will continue to emerge with the development of approaches that consider complex sociocultural variables, account for within-population heterogeneity, and avoid reference bias. With increasing genomic and phenotypic data available from populations around the world, we are only beginning to characterize genetic underpinnings of traits, the importance of their genetic background, and mechanisms of adaptation. Importantly, as we move forward, the field must emphasize the inclusion of local communities, ensure that people maintain agency over their genomic information, prioritize infrastructure for science, and improve scientific theories by collaborating across disciplinary knowledge [70–75].
